# Pediatric Eyelid Lentigo Simplex Treated With a 755‐nm Picosecond Alexandrite Laser: A Case Report

**DOI:** 10.1111/srt.70370

**Published:** 2026-07-15

**Authors:** Xin Tang, Chong Zhang, Qian Ye, Tianyi Zhang, Yan Yan, Baoxi Wang

**Affiliations:** ^1^ Department of Dermatology Plastic Surgery Hospital Chinese Academy of Medical Sciences and Peking Union Medical College Beijing China

**Keywords:** lentigo simplex, pediatric, picosecond laser

## Abstract

Lentigo simplex is a benign melanocytic lesion commonly arising in childhood. We report a 10‐year‐old girl with a stable periorbital lentigo simplex treated using a 755‐nm picosecond alexandrite laser. The patient underwent five treatment sessions over three years, resulting in significant pigment reduction without adverse effects or recurrence. This case highlights the long‐term safety and efficacy of picosecond laser treatment for pediatric facial pigmented lesions, particularly in cosmetically sensitive areas. It also adds to the limited literature supporting laser use in children and emphasizes the importance of noninvasive options for aesthetic concerns.

## 1 Case Report

Lentigo simplex is a common benign melanocytic lesion that typically arises in childhood and remains stable over time [1]. While generally isolated and asymptomatic, lesions in cosmetically sensitive areas such as the periorbital region may prompt treatment. Recent evidence suggests lentigo simplex may also appear as part of mosaic skin disorders involving keratin mutations, such as *KRT10*, indicating a possible role of keratinocyte–melanocyte interactions in pigmentation [2]. Laser treatment is commonly used to address pigmentary lesions. Picosecond‐domain alexandrite lasers offer enhanced pigment clearance with minimal thermal damage via photomechanical disruption of melanin. While picosecond lasers are widely used in pigmentary conditions [3], their long‐term safety and efficacy in treating facial melanocytic lesions in children—particularly in the sensitive periorbital region—remain underreported. Here, we report the successful use of a 755‐nm picosecond alexandrite laser in a child with stable periorbital lentigo simplex, with favorable long‐term outcomes and no adverse effects over a three‐year follow‐up.

**FIGURE 1:**
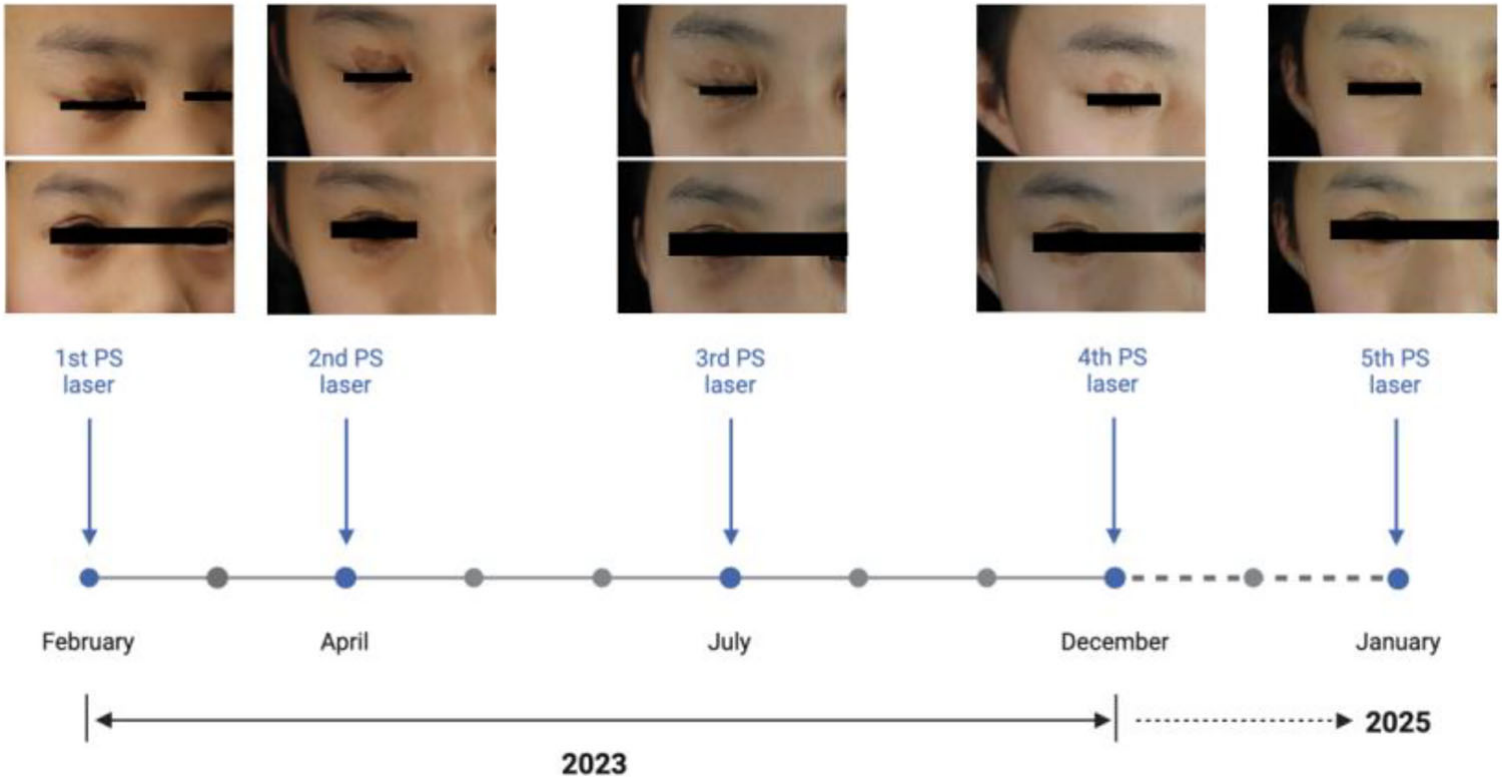
Timeline and clinical photographs showing serial picosecond (PS) laser treatments for a periorbital lentiginous nevus. Representative clinical images of the lesion site taken before and after each PS laser session are displayed from left to right, corresponding to the treatment timeline. The patient received a total of five 755‐nm picosecond alexandrite laser treatments, performed in February, April, and July of 2023, followed by additional sessions in December 2024 and January 2025. Photographs include both closed and open‐eye views to highlight changes in pigmentation and lesion contour. Progressive lightening of the lesion and cosmetic improvement were observed over time.

A 10‐year‐old girl presented with a pigmented macule around her right eye which was first noted by the patient shortly after birth. The lesion appeared as multiple, scattered brown macules—some of which were discrete, while others had coalesced into a small patch. The overall area measured approximately 3 cm in diameter, with well‐defined borders and uniform pigmentation (Figure 1). The lesion exhibited no signs of asymmetry, irregular borders, or color variation, and had remained unchanged since infancy. The diagnosis of lentigo simplex was made. Biopsy was declined because of the risk of scarring in the periorbital area.

The patient underwent five treatment sessions using a 755‐nm picosecond alexandrite laser (PicoSure; Cynosure) with a standard zoom handpiece (Figure 1). Topical 5% lidocaine cream was applied 60 min prior to treatment. During the procedure, an external lead eye shield was placed for ocular protection. Treatment parameters included a spot size of 2.5 mm and an energy density of 4.07 J/cm^2^ every time. The clinical endpoint was immediate slight whitening and mild erythema. Post‐treatment, cold packs and mupirocin ointment were applied to the lesion.

At a follow‐up visit three years after initial presentation, the lesion remained stable in size and morphology, with no evidence of peripheral extension or clinical atypia. To further reduce residual pigmentation for cosmetic reasons, a fifth laser session was performed. Post‐treatment evaluation demonstrated a marked reduction in pigmentation intensity, with no adverse effects or signs of recurrence.

Lentigo simplex is a benign melanocytic lesion that typically arises in childhood and remains clinically stable over time [4]. In this case, the lesion had been present since birth, with no appreciable changes in size, shape, or pigmentation throughout the patient's life. The classic morphology, longstanding stability, absence of atypical features, and consistent response to laser therapy provided a strong clinical basis for diagnosis.

The patient was treated using a 755‐nm picosecond alexandrite laser, which proved to be both effective and well‐tolerated in achieving pigment reduction. The treatment success observed in this case can be attributed to the unique mechanism of picosecond lasers. While both nanosecond and picosecond lasers induce pigment fragmentation, picosecond lasers utilize shorter pulse durations to generate more intense photomechanical stress [5]. This results in finer fragmentation of melanin particles into debris with minimal collateral thermal damage to surrounding tissues, thereby improving clearance while enhancing safety in sensitive areas like the eyelid. The 755‐nm wavelength was selected over 532‐nm to minimize the risk of purpura and post‐inflammatory hyperpigmentation, which is particularly important in pediatric periorbital treatments. The fluence was maintained at a stable level (4.07 J/cm^2^) throughout all sessions to prioritize safety and minimize the risk of scarring or post‐inflammatory hyperpigmentation in the delicate periorbital skin of a child.

In this patient, treatment with a non‐fractionated beam at moderate fluence resulted in visible pigment clearance, highlighting the potential clinical value of picosecond laser for benign pediatric pigmentary lesions. This case contributes to the expanding body of evidence supporting the efficacy of picosecond lasers not only in tattoo removal and melasma, but also in the management of focal pigmented lesions in younger individuals.

## Funding

This work was supported by grant from the National Key Clinical Specialty Discipline Construction Program of China (No. 23030) and Beijing Clinical Research Ward (BCRW) Project, Project Number: BCRW202202.

## Consent

Written informed consent was obtained from the patient's parents for publication of this case report and any accompanying images.

## Data Availability

Data sharing not applicable to this article as no datasets were generated or analyzed during the current study.
